# Coumarins effectively inhibit bacterial α-carbonic anhydrases

**DOI:** 10.1080/14756366.2021.2012174

**Published:** 2022-01-03

**Authors:** Simone Giovannuzzi, Chad S. Hewitt, Alessio Nocentini, Clemente Capasso, Daniel P. Flaherty, Claudiu T. Supuran

**Affiliations:** a Pharmaceutical and Nutraceutical Section, Neurofarba Department, University of Florence, Florence, Italy; b Department of Medicinal Chemistry and Molecular Pharmacology, College of Pharmacy, Purdue University, West Lafayette, IN, USA; c Department of Biology, Agriculture and Food Sciences, CNR, Institute of Biosciences and Bioresources, Napoli, Italy; d Purdue Institute for Drug Discovery, West Lafayette, IN, USA; e Purdue Institute of Inflammation, Immunology and Infectious Disease, West Lafayette, IN, USA

**Keywords:** Carbonic anhydrase, inhibitor, coumarins, *Neisseria gonorrhoeae*, antibacterials

## Abstract

Coumarins are known to act as prodrug inhibitors of mammalian α-carbonic anhydrases (CAs, EC 4.2.1.1) but they were not yet investigated for the inhibition of bacterial α-CAs. Here we demonstrate that such enzymes from the bacterial pathogens *Neisseria gonorrhoeae* (NgCAα) and *Vibrio cholerae* (VchCAα) are inhibited by a panel of simple coumarins incorporating hydroxyl, amino, ketone or carboxylic acid ester moieties in various positions of the ring system. The nature and the position of the substituents in the coumarin ring were the factors which strongly influenced inhibitory efficacy. NgCAα was inhibited with K_I_s in the range of 28.6–469.5 µM, whereas VchCAα with K_I_s in the range of 39.8–438.7 µM. The two human (h)CA isoforms included for comparison reason in the study, hCA I and II, were less prone to inhibition by these compounds, with K_I_s of 137–948.9 µM for hCA I and of 296.5–961.2 µM for hCA II, respectively. These findings are relevant for discovering coumarin bacterial CA inhibitors with selectivity for the bacterial over human isoform, with potential applications as novel antibacterial agents.

## 1. Introduction

**Figure 1.**
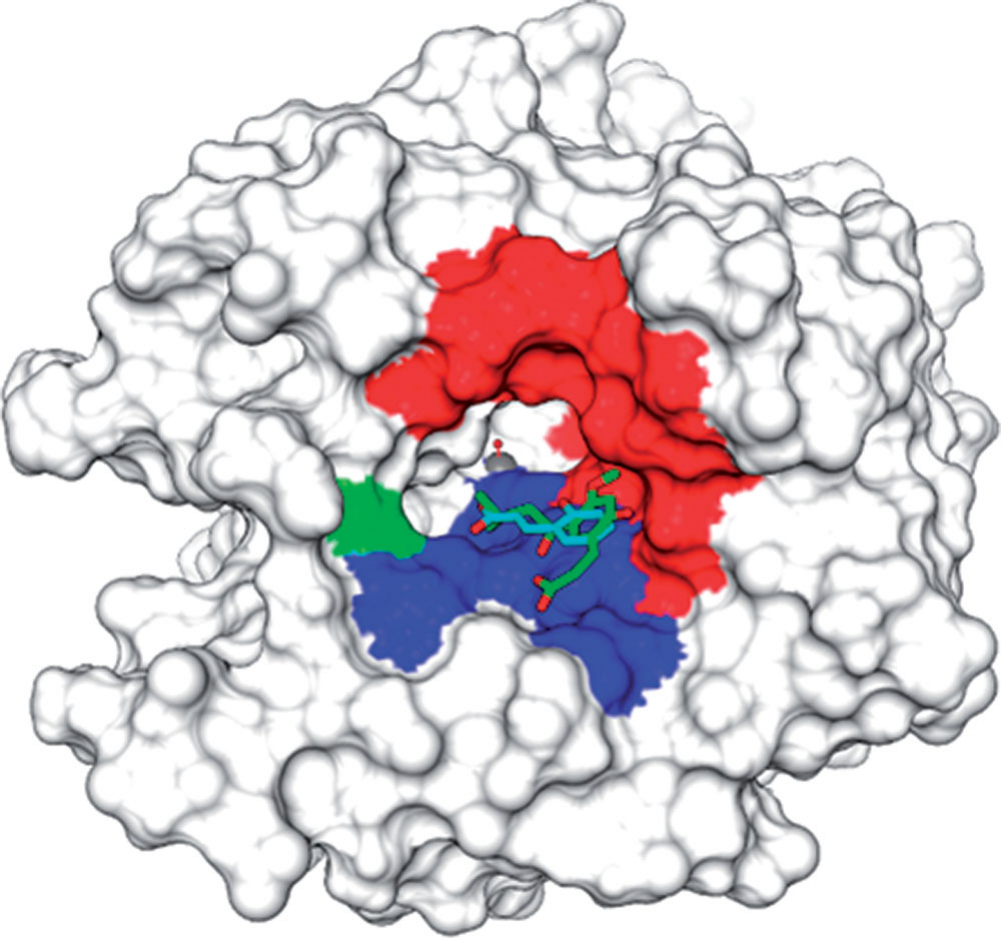
Surface representation of hCA II in adduct with the superimposed hydrolized (and active) coumarin species (cyan from 5BNL, green from PDB 3F8E). The hydrophobic half of the active site is coloured in red, the hydrophilic one in blue. His64, the proton shuttle residue, is in green.

Bacterial genomes encode for at least four genetic families of the enzyme carbonic anhydrase (CA, EC 4.2.1.1), the α-, β-, γ- and ι-CAs^1–3^. These enzymes catalyse the interconversion between CO_2_ and bicarbonate, generating H^+^ ions which have a role in pH regulation processes in prokaryotes and eukaryotes^3–8^. However, CAs possess other crucial functions in bacteria, participating in metabolic processes that encompass carboxylating reactions in which both CO_2_ and bicarbonate may act as substrates^2^^,^^3^^,^^9^, but also in photosynthesis in the case of cyanobacteria^9^. These relevant functions that CAs play in bacteria led to the proposal of using their inhibitors as novel antibacterial agents, considering the well-known and prevalent phenomenon of drug resistance to clinically used antibiotics^2–7^. In fact, relevant inhibition of growth has been reported for several pathgenic bacteria (e.g. *Helicobacter pylori*^10^, vancomycin-resistant enterococci^4^, *Neisseria gonorrheae*^4^, etc.) with sulphonamides, the most investigated class of CA inhibitors (CAIs)^11^. However, there are many other classes of CAIs, which possess a rather diversified mode of action and inhibition mechanisms in human CAs compared to sulphonamides^12^, yet these have been scarcely investigated for the inhibition of bacterial CAs. One such class of CAIs is represented by the coumarins (and their derivatives) ^13–15^, which have been shown to be mechanism-based suicide (prodrug) inhibitors. For example, the esterase activity of CAs appears to hydrolyse the lactone ring of coumarins to generate 2-hydroxy-cynnamic acids which bind at the entrance of the CA active site^13^, as shown in Figure 1.

Many coumarins proved to act as efficient and also isoform-selective CAIs^13–15^ targeting the 15 mammalian CAs known to date^11^^,^^12^, due to the fact that they bind at the entrance of the active site, where the highest variability in the composition of amino acid residues is found in the different human (h)CA isoforms^11^^,^^12^. However, as mentioned above no bacterial α-CAs have been investigated until now for their interaction with coumarins, and this gap in the field is filled here by our report that a small panel of simple coumarin derivatives indeed inhibit two bacterial α-class enzymes from two human pathogens of urgent concern, *Vibrio cholerae* and *Neisseria gonorrheae*. It should be mentioned that the esterase activity of CAs is only documented and investigated in detail for the α-class of CAs. We have shown previously that β-, δ- and γ-CAs (also present in some bacteria) do not possess esterase activity^16^, whereas for other genetic CA families only scarce or inconclusive data are available in the literature^17^^,^^18^. Thus, due to the lack of esterase activity observed in other bacterial CA isoforms we chose to investigate only bacterial α-CAs for their possible inhibition with coumarins.

## 2. Materials and methods

### 2.1. Enzymology and CA activity and inhibition measurements

An Applied Photophysics stopped-flow instrument was used to assay the CA-catalysed CO_2_ hydration activity^19^. Phenol red (0.2 mM) was used as a pH indicator, working at the absorbance maximum of 557 nm, with 10 mM HEPES (pH 7.4) as a buffer, and in the presence of 10 mM NaClO_4_ to maintain constant ionic strength, in order to follow the initial rates of the CA-catalysed CO_2_ hydration reaction for a period of 10–100 s. The CO_2_ concentrations ranged from 1.7 to 17 mM for the determination of the kinetic parameters and inhibition constants. For each inhibitor, at least six traces of the initial 5–10% of the reaction were used to determine the initial velocity. The uncatalyzed rates were determined in the same manner and subtracted from the total observed rates. Stock solutions of inhibitors (10–20 mM) were prepared in distilled-deionized water, and dilutions up to 10 nM were done thereafter with the assay buffer. Inhibitor and enzyme solutions were preincubated together for 1–6 h at 4 °C prior to the assay, in order to allow for the formation of the E-I complex. The inhibition constants were obtained by non-linear least-squares methods using Prism 3 and the Cheng-Prusoff equation, as reported previously^13–15^, and represent the mean from at least three different determinations. The NgCAα concentration in the assay system was 6.8 nM whereas the VchCAα was 9.2 nM. The used enzymes were recombinant proteins obtained in-house, as described earlier^5^^,^^6^.

### 2.2. Chemistry

Coumarins **1–14**, buffers, acetazolamide **AAZ** and other reagents were of > 99% purity and were commercially available from Sigma-Aldrich (Milan, Italy).

## 3. Results and discussion

Bacterial CAs were thoroughly investigated for their inhibition with the two main types of classical CAIs, the sulphonamides (and their isosteres) and the metal complexing anions^2–8^. However, no inhibition data with the many other classes of inhibitors, including the coumarins, are available so far in the literature for these enzymes^11^^,^^12^.

Thus, we decided to investigate a series of simple coumarin derivatives of type **1–14** (Table 1) with their interaction with two bacterial α-CAs, NgCAα and VChCAα, for which sulphonamide/anion inhibition data have already been reported in the literature^5^^,^^6^. Both enzymes have been proposed as potential antibacterial drug targets and their inhibitors might be useful to address the antibiotic drug resistance which constitutes a serious medical problem worldwide^2^. Being the first investigation of coumarins as potential bacterial CAIs, we have chosen relatively simple scaffolds in order to delineate the structure-activity relationship (SAR) of this underexplored class of CAI. As seen from Table 1, the simple unsubstituted coumarin **1** as well as its mono- and di-substituted derivatives in various positions of the ring system were included in our study. The moieties present in these derivatives were again rather simple but derivatizable ones, such as hydroxyl, primary/tertiary amino, ketone, carboxylic acid ester, and they are found in diverse combinations and positions on the ring system. In fact, we have demonstrated earlier, for mammalian CAs, that the nature of these moieties and the substitution pattern on the coumarin ring are the most prominent features connected with efficient inhibitory action^13–15^.

**Table 1.** Inhibition data of hCA I and II and bacterial enzymes NgCAα and VchCAα using AAZ as standard drug by a stopped-flow CO_2_ hydrase assay at 6 h incubation time between enzyme and inhibitor.

Name	Structure	*K_i_* (µM)^a^			
		hCA I	hCA II	NgCAα	VchCAα
1	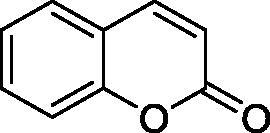	160.0 (3.1)^b^	600.0 (9.2)^b^	81.6	94.7
2	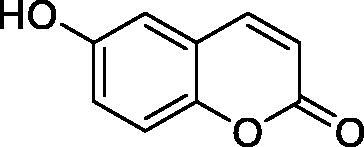	192.0	683.0	92.4	77.5
3	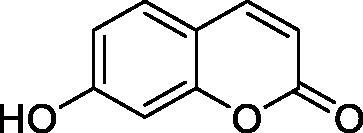	263.5	690.6	77.1	68.5
4	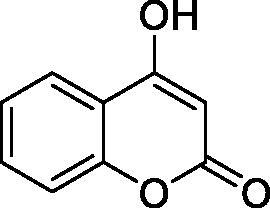	393.5	513.1	94.7	92.2
5	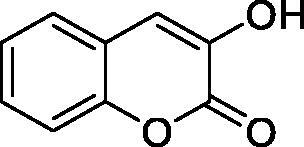	489.8	625.2	110.0	289.5
6	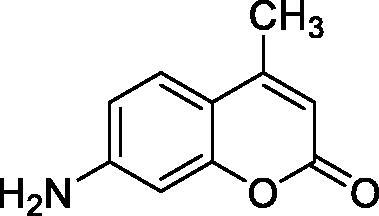	646.3	485.7	70.9	71.1
7	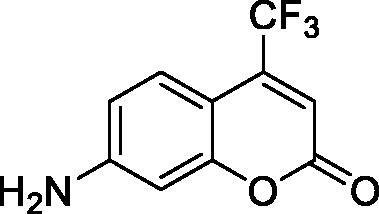	939.6	733.5	97.1	95.0
8	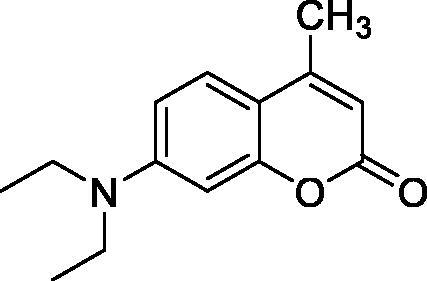	516.5	558.9	28.6	53.9
9	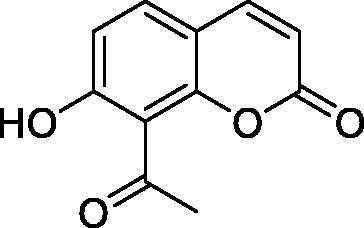	948.9	646.2	42.5	39.8
10	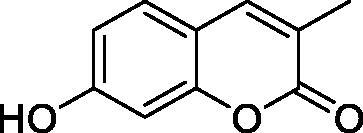	137.0	296.5	68.0	66.8
11	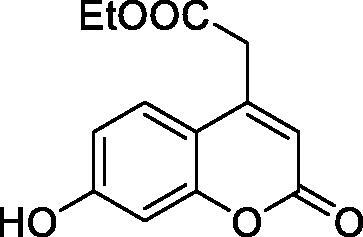	748.9	875.6	469.5	438.7
12	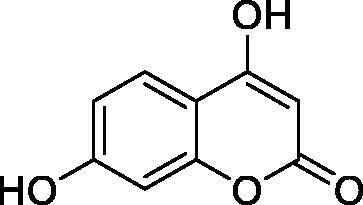	181.8	758.4	77.6	66.0
13	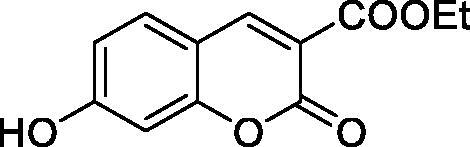	900.1	961.2	394.5	431.9
14	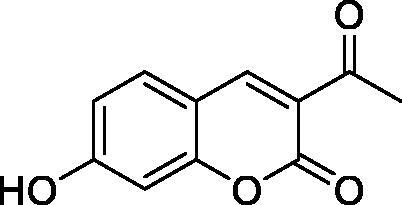	469.7	786.2	394.5	302.7
AAZ	–	0.25	0.012	0.075	0.0068

^a^Mean from three different assays, by a stopped flow technique (errors were in the range of ± 5–10% of the reported values); ^b^Data from ref.^13^, with a longer incubation time between enzyme and coumarin.

We observed that as for the mammalian CAs for which coumarins were reported to act as inhibitors^13–15^, the inhibition process of bacterial enzymes is different compared to inhibition with sulphonamides/anions that was reported previously^2–5^. For sulphonamide inhibitors the rapid equilibration between the enzyme and inhibitor to form the enzyme-inhibitor complex typically is achieved in a few minutes, thus, this is the reason why the enzyme and the sulphonamide/anion inhibitors are generally incubated for 15 min prior to assay^2^^,^^5^^,^^19^. However, for coumarins and mammalian CAs, which require catalytic cleavage of the lactone ring prior to the enzyme-inhibitor complex being formed, such an inhibition period led to weak millimolar inhibitory activity for a range of structurally diverse coumarins^13^. For this reason, the inhibition was investigated with longer incubation times, of 1–24 h, which led to the observation that the process is time-dependent, with an inhibitory action increasing over time, and typically an equilibrium is achieved after 6 h incubation between enzyme coumarin^13^. X-ray crystallography and detailed kinetic measurements thereafter confirmed the fact that the coumarin is hydrolysed by the esterase CA activity leading to the formation of the 2-hydroxy-cinnamic acids shown in Figure 1, which in fact are the *de facto* CAIs. The same situation was observed here for the inhibition of the two investigated bacterial enzymes with coumarins **1–14**: a time dependency of the inhibition has been observed, with a steady inhibitory effect being achieved after 6 h incubation of the enzymes and the coumarin (data not shown). Thus, all coumarins were investigated as CAIs in the same conditions as for hCAs, and data of Table 1 report K_I_s obtained after 6 h incubation time. The following SAR can be drawn from data of Table 1:

- All coumarins **1–14** inhibited the two bacterial enzymes with inhibition constants in the medium–high micromolar range. The modest potency is not unexpected considering the simple structures, but as our intention was to provide a proof-of-concept study that bacterial CAs are inhibited by non-sulphonamide compounds, the modest inhibition was acceptable. For NgCAα the K_I_s were in the range of 28.6–469.5 µM, whereas for VchCAα in the range of 39.8–438.7 µM. It should be observed that the two human isoforms included for comparison reason in the study, hCA I and II, were also weakly inhibited by these compounds, as the K_I_s were in the range of 137–948.9 µM for hCA I and of 296.5–961.2 µM for hCA II, respectively. It is in fact well-known that the cytosolic hCAs show a poor inhibitory effect with coumarins (as also reconfirmed here) whereas many trans-membrane, tumor-associated CAs, such as hCA IX and XII, lead in many cases to low nanomolar inhibitors^20^.
- The substitution pattern of the coumarin seems to be the most relevant factor connected with inhibitory efficacy. This was observed for both bacterial CAs investigated here and the panel of coumarins **1–14**, similar to what was previously reported for hCAs^13–15^. Thus, substituents bulkier than H or Me in position 3, led to ineffective bacterial CAIs (e.g. coumarins **13** and **14** against both bacterial enzymes; **5** against VchCAα), the same was also observed for **11**, possessing a bulky group in position 4, which was the least effective coumarin against bacterial CAs in the investigated series. Smaller and more compact moieties in position 4, such as OH (compounds **4** and **12**), methyl (compounds **6** and **8**), CF_3_ (derivative **7**) were tolerated and led to effective micromolar inhibitors (Table 1).
- Substituents in positions 6, 7 and/or 8 of the coumarin ring generally led to effective CAIs against both bacterial enzymes, a situation also observed for hCA IX/XII; as those groups do not interfere with the hydrolysis of the lactone ring, being further away from carbonyl site of hydrolysis^13–15^. Thus, 4-methyl-7-diethylamino-coumarin **8** was the most effective NgCAα inhibitor in the series (K_I_ of 28.6 µM) whereas 7-hydroxy-8-acetyl-coumarin **9** was the most effective VchCAα inhibitor (K_I_ of 39.8 µM). With respect to placement of the substituents on the coumarin rings there are a couple factors to consider regarding potency. First, steric hinderance by substituents nearby the hydrolysable bond of the lactone may reduce the ability of the CA to cleave the ester. Second, there may be electronic considerations with the substituents they could make the carbonyl more electrophilic for attack by water to provide the corresponding cinnamic acids. Alternatively, these substituents may also be involved in the binding interaction of the resulting cinnamic acids, and either improve inhibition of reduce it.
- A range of the tested coumarins had a behaviour of medium potency inhibitors against both bacterial isoforms, with K_I_ values < 100 µM, being thus amenable to be considered as viable hit compounds for developing tighter binding compounds. Indeed, some of these derivatives such as **2, 3, 4, 6, 7, 9, 10**, and **12** incorporate free OH or NH_2_ moieties which are easy to derivatize in a multitude of ways, and in the case of hCAs led to much more effective CAIs compared to the lead^21^.
- The sulphonamide CAI acetazolamide (AAZ) used as standard compound was much more effective for the inhibition of the bacterial enzymes as expected. However, an interesting observation is that some of the coumarins investigated here do show a much better inhibitory profile for the bacterial over the human isoforms (e.g. **8** and **9**), which may prove beneficial for obtaining potential antibacterials with selectivity over human isoforms, such as hCA I and II.

## 4. Conclusions

This is the first report demonstrating that bacterial α-class CAs are susceptible to inhibition by coumarins, a class of inhibitors investigated previously only for their interaction with human CA isoforms. Our data indicate that a panel of simple coumarin derivatives inhibit two enzymes from human bacterial pathogens with a medium efficacy in the low–medium micromolar range. This proof-of-concept study demonstrates that coumarins possess inhibitory potential and lays groundwork to further explore SAR modifications to probe steric and electronic contributions to bacterial CA hydrolysis of the lactone prodrug and/or contributions to binding and inhibition of the resulting cinnamic acids. Additionally, significant selectivity for inhibiting the bacterial over the human isoforms hCA I and II was observed suggesting promising data for obtaining more effective and bacterial CA–selective coumarin inhibitors. Bacterial CA active sites are slightly more voluminous compared to the mammalian CA isoforms active sites^10^, which may explain why bacterial enzymes are more effectively inhibited by this class of compounds. Indeed, many of the effective bacterial CA inhibitory coumarins incorporate easily derivatizable moieties of the phenol, amine, ketone or carboxylate type, which in principle can be used for obtaining better inhibitors. This investigation warrants further studies in order to find effective non-sulphonamide CAIs which may be useful for exploring antibacterials that can revert the extensive drug resistance observed with the clinically used anytibiotics.
